# Laparoscopic Surgery Is Associated With Reduced Small Bowel Obstruction Risk After Colorectal Cancer Surgery: A Nationwide Cohort Study of 5458 Patients

**DOI:** 10.1002/ags3.70280

**Published:** 2026-09-10

**Authors:** Takeshi Yamada, Fumihiko Fujita, Ken Eto, Kozo Kataoka, Norio Yukawa, Kiichi Sugimoto, Rai Shimoyama, Atsuko Fukazawa, Kensuke Kumamoto, Yuichi Takayama, Akira Komono, Akihisa Matsuda, Ryo Ohta, Hiromichi Sonoda, Koichi Okuya, Keisuke Ihara, Yasuyuki Yokoyama, Takuya Nishino, Yasuki Akiyama, Daisuke Ichikawa

**Affiliations:** ^1^ Department of Gastroenterological Surgery Nippon Medical School Tokyo Japan; ^2^ Department of Surgery Kurume University School of Medicine Kurume Japan; ^3^ Department of Surgery The Jikei University School of Medicine Tokyo Japan; ^4^ Division of Lower GI, Department of Gastroenterological Surgery Hyogo Medical University Nishinomiya Japan; ^5^ Department of Surgery Yokohama City University Yokohama Japan; ^6^ Department of Coloproctological Surgery Juntendo University Faculty of Medicine Tokyo Japan; ^7^ Department of General Surgery Saitama Memorial Hospital Saitama Japan; ^8^ Department of Gastroenterological Surgery Iwata City Hospital Iwata Japan; ^9^ Department of Genome Medical Science and Medical Genetics, Faculty of Medicine Kagawa University Kagawa Japan; ^10^ Department of Surgery Ogaki Municipal Hospital Ogaki Japan; ^11^ Department of Gastroenterological Surgery Fukuoka University Faculty of Medicine Fukuoka Japan; ^12^ Department of Surgery JR Sapporo Hospital Sapporo Japan; ^13^ Department of Colorectal Surgery Dokkyo Medical University Tochigi Japan; ^14^ Department of Health Care Administration Nippon Medical School Tokyo Japan; ^15^ Department of Surgery 1, School of Medicine University of Occupational and Environmental Health Fukuoka Japan; ^16^ First Department of Surgery Yamanashi University Kofu Japan

**Keywords:** adhesion prevention material, colorectal cancer, laparoscopic surgery, small bowel obstruction, stoma

## Abstract

**Introduction:**

Postoperative small bowel obstruction (SBO) is a major complication following colorectal cancer surgery, yet evidence‐based prevention strategies remain unclear. We aimed to clarify site‐specific risk factors for SBO and evaluate the effectiveness of laparoscopic surgery and adhesion prevention materials (APMs) in preventing SBO after colorectal cancer surgery.

**Methods:**

This retrospective cohort study analyzed 5458 patients who underwent colorectal cancer surgery at 32 Japanese institutions between 2012 and 2014. The primary endpoint was the 5‐year risk of SBO. We evaluated the effects of laparoscopic surgery, APM use, and stoma creation on SBO risk. Clinical variables included demographics, tumor site, operative approach, operative details, and postoperative complications. Hospital‐level clustering was addressed using mixed‐effects logistic regression.

**Results:**

Overall SBO incidence was 5.2% (*n* = 283). Rectal cancer had the highest risk, whereas all colonic sites except the descending colon showed significantly lower odds. Laparoscopic surgery was associated with a 42% reduction in odds (OR 0.58; 95% CI 0.45–0.74; *p* < 0.001), with significant reductions in ascending (NNT = 22.2, *p* = 0.001) and sigmoid colon surgery (NNT = 30.2, *p* = 0.003). APMs showed no protective effect (OR 1.01; 95% CI 0.78–1.32; *p* = 0.94). Stoma creation significantly increased SBO risk (OR 1.84; 95% CI 1.35–2.51; *p* < 0.001). Secondary analysis identified reoperation and postoperative ileus as additional independent risk factors.

**Discussion:**

Laparoscopic surgery was associated with reduced long‐term SBO risk, with significant benefits in ascending and sigmoid colon surgery. APMs showed no measurable benefit. Stoma creation increased SBO risk, with no observed difference between ileostomy and colostomy.

## Abbreviated Abstract

This nationwide retrospective cohort study of 5458 colorectal cancer surgeries across 32 Japanese institutions examined the effects of laparoscopic surgery, adhesion prevention materials (APMs), and stoma creation on the 5‐year risk of small bowel obstruction (SBO). Laparoscopic surgery was independently associated with a 42% reduction in SBO risk (OR 0.58; 95% CI 0.45–0.74; *p* < 0.001), with statistically significant reductions observed in ascending and sigmoid colon surgery, whereas APMs showed no protective effect (OR 1.01; 95% CI 0.78–1.32; *p* = 0.94). Stoma creation was identified as a major independent risk factor (OR 1.84; 95% CI 1.35–2.51; *p* < 0.001), underscoring the need for judicious patient selection when planning stoma formation.

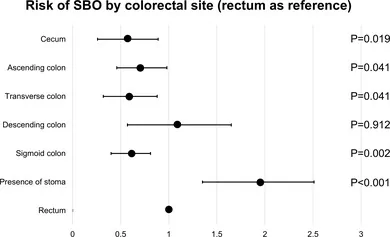

## 1 Introduction

Postoperative small bowel obstruction (SBO) is a major complication following colorectal cancer surgery, accounting for 12%–16% of surgical admissions in the United States with substantial healthcare costs [1]. This burden is also evident worldwide, as shown in Swedish and Korean registries [2, 3]. SBO management is often prolonged and may require reoperation or extensive bowel resection, resulting in impaired nutrition, reduced quality of life, and increased morbidity [4]. Moreover, operative mortality approaches 5% [5]. Despite this clinical and economic burden, standardized prevention strategies remain lacking. Current practice depends largely on surgeon preference, and high‐quality evidence for effective preventive interventions is insufficient.

Postoperative SBO primarily results from adhesion formation due to parietal and visceral peritoneal damage. Laparoscopic surgery minimizes peritoneal trauma, while adhesion prevention materials (APMs) provide physical barriers between damaged peritoneum. While some randomized trials in general or gynecological surgery have shown a reduction in adhesion formation [6, 7, 8], evidence for preventing clinically significant SBO after colorectal cancer surgery remains inconsistent. Several observational studies have suggested lower rates of SBO with laparoscopic compared to open surgery [3, 9, 10]. However, randomized controlled trials, including the COLOR II trial of rectal cancer patients, failed to demonstrate a significant reduction in SBO with laparoscopic surgery [11]. Although laparoscopic resection reduces parietal adhesions, it does not effectively prevent visceral adhesions, which may explain its limited efficacy in SBO prevention [12]. Neither APMs nor laparoscopic surgery has been definitively established as reliable SBO prevention strategies, highlighting the need for disease‐specific evaluation.

To clarify these uncertainties, we previously conducted a nationwide survey of 18 798 gastrointestinal surgeries across 32 institutions [13]. That study demonstrated that laparoscopic procedures reduced SBO incidence in colorectal and gastroduodenal surgery, while APMs showed no benefit. In a separate cohort of 2058 patients with SBO, postoperative colorectal surgery accounted for approximately one‐third of cases (*n* = 661). However, that analysis included diverse gastrointestinal diseases (inflammatory bowel disease, diverticular disease) without focusing specifically on colorectal cancer and lacked important subgroup evaluations such as site‐specific differences or stoma impact. Given that colorectal cancer was the predominant cause, a disease‐specific analysis was warranted.

These conflicting results underscore two critical unresolved issues: (i) whether laparoscopic surgery and APMs truly prevent clinically meaningful SBO in colorectal cancer patients, and (ii) whether anatomical site or stoma creation confers distinct risks. To address these gaps, we performed a large, disease‐specific nationwide analysis.

## 2 Methods

### 2.1 Study Design and Ethical Considerations

This study represents a post hoc analysis of a nationwide retrospective cohort conducted by the Japanese Society for Abdominal Emergency Medicine between 2012 and 2014. Ethical approval was obtained from the local Institutional Review Board (Approval No. 29–01‐887), and the study was carried out in accordance with the Declaration of Helsinki. An opt‐out procedure for informed consent was applied at all participating institutions.

### 2.2 Patient Selection and Data Collection

During the study period, 18,798 gastrointestinal surgeries were performed across 32 hospitals, of which 5811 involved colorectal disease. Patients were eligible if they had histologically confirmed colorectal cancer, while those with benign conditions (e.g., diverticulitis, inflammatory bowel disease) or incomplete clinical data (e.g., surgical approach, postoperative course) were excluded. The primary endpoint was the 5‐year risk of SBO, defined as a binary outcome indicating whether SBO occurred within five years after surgery. Although described as a cumulative incidence measure, the analysis evaluated SBO occurrence within the 5‐year follow‐up period using mixed‐effects logistic regression rather than time‐to‐event models. The retrospective survey questionnaire was designed to capture SBO occurrence as a binary variable (yes/no within five years), and the exact date of SBO onset was not recorded; consequently, time‐to‐event analysis could not be performed.

Clinical variables collected included patient demographics (age, sex), tumor location, operative approach (laparoscopic or open), stoma type and site (if present), use of APMs, operative time, estimated blood loss, prophylactic drain placement, and postoperative outcomes including ileus, anastomotic leakage, intra‐abdominal abscess, reoperation, and SBO within five years. During the study period, only Seprafilm was approved for gastrointestinal surgery in Japan, and its use and application site were determined at the surgeon's discretion without standardization, introducing potential indication bias. SBO was defined as clinically diagnosed SBO requiring hospitalization and fasting for more than one day, with cases suspected of cancer recurrence excluded. Both cases that improved with conservative management and those requiring surgical intervention were included.

The dataset did not capture information on peritoneal dissemination, combined resection of other organs, or emergency surgery, and such cases were therefore not excluded. The potential inclusion of these cases represents an important limitation of this study. In addition, other important confounders such as diabetes mellitus, body mass index, TNM staging, and details of neoadjuvant therapy were not available, further limiting the dataset.

### 2.3 Statistical Analysis

Missing data were excluded by listwise deletion. Categorical variables were compared using the Fisher's exact test.

The primary analytical approach employed mixed‐effects logistic regression models with hospital as a random intercept to account for institutional clustering effects. Funnel plot analysis was performed to visualize inter‐institutional variation in SBO incidence. To avoid unstable estimates arising from very small case volumes, funnel plot analysis was limited to institutions with ≥ 20 cases. This model evaluated associations between surgical approach (laparoscopic vs. open), APM use, and the occurrence of SBO within five years. Fixed effects included age (continuous, centered), sex, tumor location, and stoma creation. Model performance was assessed using the Akaike Information Criterion (AIC) and Bayesian Information Criterion (BIC), with ΔAIC > 2 and ΔBIC > 6 considered meaningful differences [14]. The intraclass correlation coefficient (ICC) was calculated to quantify the proportion of variance attributable to between‐hospital differences. To quantify the clinical significance of the interventions, we calculated the number needed to treat (NNT) and absolute risk reduction (ARR) for the overall analysis and subgroup analyses by tumor location for both laparoscopic surgery and APM use. In accordance with established statistical practice, NNT and ARR values were calculated only for statistically significant results (*p* < 0.05), as these metrics derived from non‐significant differences may be misleading and lack clinical interpretability. For non‐significant comparisons, only odds ratios, 95% confidence intervals, and *p*‐values are reported. Exploratory subgroup analyses stratified by tumor location and rectal cancer cases with/without stoma were conducted without adjustment for multiple comparisons.

Selection bias was inherent in this observational design, as the choice of laparoscopic surgery and APM use was not randomized but determined by surgeon preference and patient‐specific factors, potentially confounding the observed associations. To assess the potential impact of unmeasured confounding, we calculated E‐values for the associations between surgical approach, APM use, stoma creation, and SBO. The E‐value represents the minimum strength of association, on the risk ratio scale, that an unmeasured confounder would need to have with both the exposure and the outcome to fully explain away the observed association, conditional on the measured covariates.

As a secondary analysis, standard multivariable logistic regression (without random effects) was performed to identify independent SBO risk factors and to facilitate comparison with previous literature. Covariates were selected based on prior studies [15, 16, 17, 18] and included: sex, surgical approach, APM use, stoma creation, blood loss, operative time, reoperation, anastomotic leakage, and postoperative ileus.

All statistical analyses were performed using R software (version 4.3.0, R Foundation for Statistical Computing, Vienna, Austria). Mixed‐effects logistic regression models were fitted using the glmer() function in the lme4 package (version 1.1–34) [19]. Statistical significance was set at *p* < 0.05.

## 3 Results

### 3.1 Patients

There were 5811 cases of colorectal disease, of which 5458 met the inclusion criteria (259 cases of benign disease and 94 cases with insufficient data). Of these, 3193 patients (58.5%) underwent laparoscopic surgery, and 2265 (41.5%) underwent open surgery. A total of 283 cases of SBO were identified. The details of the patients are shown in Table 1. Compared with transverse colon surgery, laparoscopic surgery was significantly more frequently performed in all colorectal sites except for descending colon surgery (Figure 1A). Among all sites, the sigmoid colon had the lowest rate of APM use, with higher usage observed in every other location (Figure 1B). All colonic tumor locations showed significantly lower odds of SBO compared to rectal cancer, with the exception of descending colon (Figure 1C). The presence of a stoma was associated with an 84% increase in the odds of developing SBO (Figure 1C).

**FIGURE 1A:**
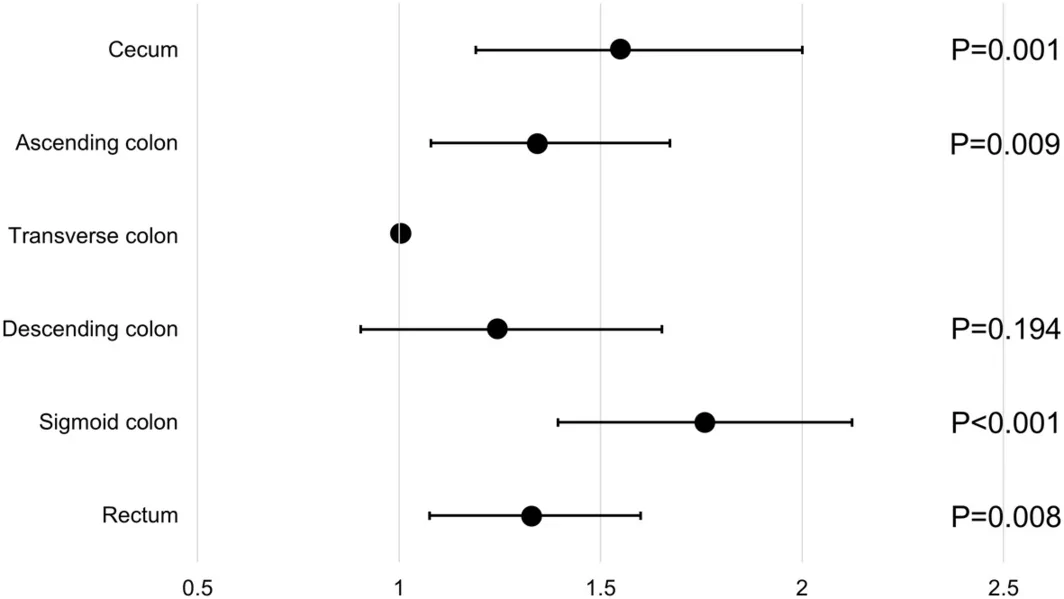
Proportion of laparoscopic surgeries (Transverse colon as reference). The proportion of laparoscopic surgery was lowest in transverse colon surgery and was significantly higher in all other colorectal sites except for the descending colon.

**TABLE 1:** Patient background by colorectal site.

*N* = 5458	Cecum (*N* = 448)	A‐colon (*N* = 953)	T‐colon (*N* = 489)	D‐colon (*N* = 263)	S‐colon (*N* = 1290)	Rectum (*N* = 2015)
Laparoscopic surgery (%)	61.2	57.8	50.5	55.5	63.7	57.2
APM (%) ALL	60.0	59.3	63.1	60.8	54.0	60.3
Laparoscopic	56.9	51.9	54.4	54.8	49.6	54.1
Open	64.9	69.4	71.9	68.4	61.5	68.4
Blood loss (mL)	40	60	60	100	30	100
Surgical time (min)	185	195	205	235	205	264
Prophylactic drain (%)	54.0	62.5	65.2	77.9	82.2	95.0
Re‐operation (%)	2.7	2.6	2.9	7.2	2.2	7.0
Anastomotic leakage (%)	2.7	3.3	2.5	8.7	9.6	26.6
Abscess formation (%)	2.2	3.1	3.5	3.8	1.4	5.9
Ileus (%)	3.3	5.7	6.3	6.8	5.7	6.2
SBO (%)	3.8	4.6	3.7	6.5	3.9	6.8

Abbreviations: APM, adhesion prevention material; SBO, small bowel obstruction.

The mixed‐effects model demonstrated good fit (AIC = 2156.3; BIC = 2231.7). The hospital‐level random intercept indicated significant variability in SBO incidence (variance = 0.0010, SD = 0.032), with an intraclass correlation coefficient of 0.031, meaning that 3.1% of the variance in SBO occurrence was explained by between‐hospital differences. This inter‐institutional variation was further illustrated by the funnel plot analysis, which revealed SBO rates ranging from 0.4% to 11.3% among the 28 institutions with ≥ 20 cases (Figure 2). Institutions with smaller volumes showed wider confidence intervals, reflecting the anticipated variability associated with limited sample sizes. In contrast, high‐volume institutions were more tightly clustered around the overall mean incidence of 5.16%. This variation underscored the importance of accounting for hospital‐level clustering in our mixed‐effects model. Our analysis did not demonstrate a clear volume‐outcome relationship for SBO prevention. Some high‐volume institutions (> 300 cases) exhibited SBO rates both above and below the overall mean.

**FIGURE 1B:**
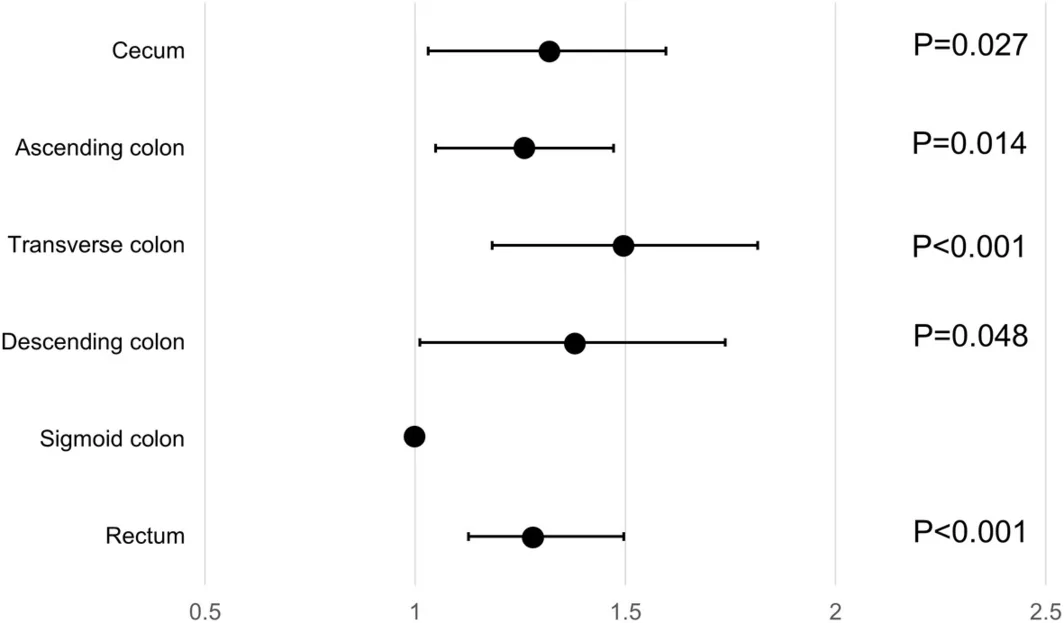
Usage rate of adhesion prevention material. Among all sites, the sigmoid colon had the lowest rate of APM use, with higher usage observed in every other location.

### 3.2 Impact of Laparoscopic Surgery on SBO Incidence

In the mixed‐effects logistic regression model, laparoscopic surgery was independently associated with a lower risk of postoperative SBO compared with open surgery (Table 2, adjusted OR 0.58, 95% CI 0.45–0.74, *p* < 0.001), corresponding to a 42% reduction in odds. To quantify the clinical significance of laparoscopic surgery, we calculated the NNT based on the ARR. Overall, laparoscopic surgery demonstrated an ARR of 2.70% compared to open surgery, yielding an NNT of 37.0. In all sections except the transverse colon, the incidence rate of SBO was lower in laparoscopic surgery compared to open surgery. However, a significant difference was observed only in the ascending colon (*p* = 0.001) and the sigmoid colon (*p* = 0.003). Among sites with statistically significant reductions, the ascending colon demonstrated an NNT of 22.2 and the sigmoid colon an NNT of 30.2.

**FIGURE 1C:**
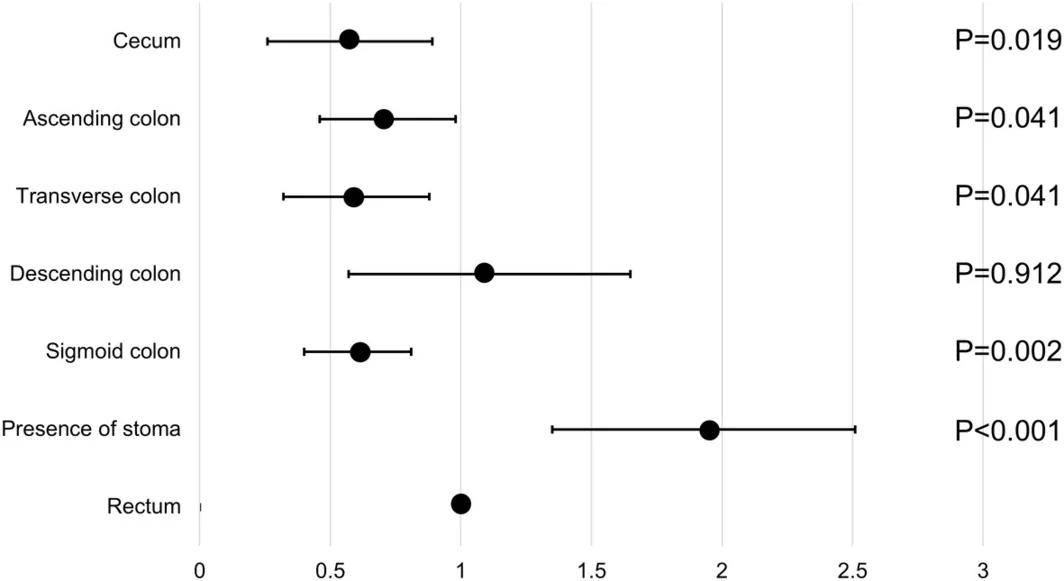
Risk of SBO by colorectal site (rectum as reference). All colonic tumor sites demonstrated significantly lower odds of SBO compared with rectal cancer, except for the descending colon. The risk associated with stoma creation was assessed relative to cases without a stoma, and the presence of a stoma was associated with an 84% increase in the odds of developing SBO (OR 1.84; 95% CI 1.35–2.51; *p* < 0.001). Results are derived from mixed‐effects logistic regression.

**TABLE 2:** Impact of laparoscopic surgery on SBO incidence.

	Laparo	Open	OR	95% CI	*p*	ARR (%)	NNT	95% CI
Total (*N* = 5458)	130 (4.1)	153 (6.8)	0.58	0.45–0.74	< 0.001	2.7	37.0	28.2–54.7
Cecum (*N* = 448)	7 (2.6)	10 (5.7)	0.51	0.17–1.54	0.14	N.A.	N.A.	N.A.
Ascending colon (*N* = 953)	15 (2.7)	29 (7.2)	0.36	0.19–0.68	0.001	4.5	22.2	15.3–38.5
Transverse colon (*N* = 489)	10 (4.0)	8 (3.3)	1.21	0.47–3.13	0.85	N.A.	N.A.	N.A.
Descending colon (*N* = 263)	6 (4.1)	11 (9.4)	0.41	0.15–1.14	0.14	N.A.	N.A.	N.A.
Sigmoid colon (*N* = 1290)	22 (2.7)	28 (6.0)	0.43	0.24–0.76	0.003	3.3	30.2	19.5–67.5
Rectum (*N* = 2015)	70 (6.1)	67 (7.8)	0.75	0.53–1.07	0.13	N.A.	N.A.	N.A.

*Note:* Each number represents the number of cases of small bowel obstruction. The values in parentheses indicate percentages.

Abbreviations: 95% CI, 95% confidence interval; APM, adhesion prevention material; ARR, absolute risk reduction; Laparo, laparoscopic; N.A, not availableNNT, number needed to treat; OR, odds ratio; SBO, small bowel obstruction.

**FIGURE 2:**
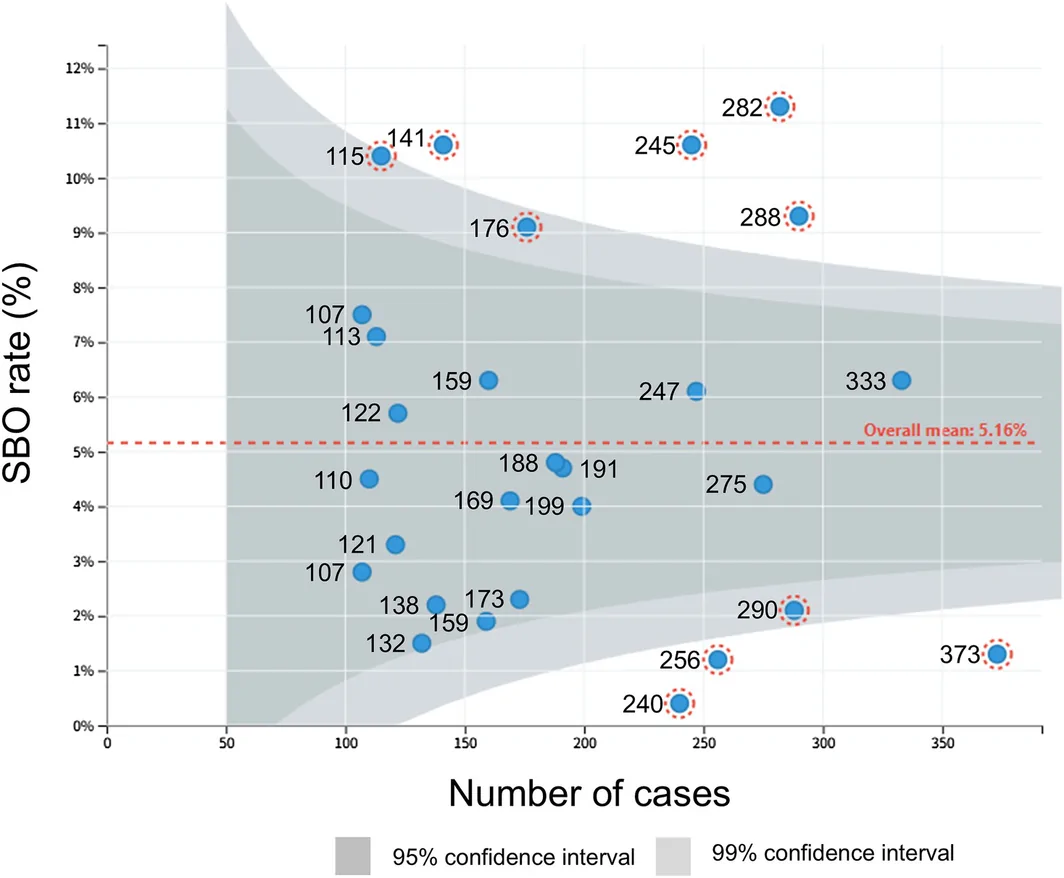
Funnel plot analysis of SBO outcomes across institutions. The numbers next to the dots indicate the number of surgeries. The funnel plot analysis demonstrated considerable inter‐institutional variation in SBO rates, ranging from 0.4% to 11.3% among the 28 institutions with ≥ 20 cases. Institutions with smaller volumes showed wider confidence intervals, reflecting the anticipated variability associated with limited sample sizes. In contrast, high‐volume institutions were more tightly clustered around the overall mean incidence of 5.16%. Our analysis did not demonstrate a clear volume‐outcome relationship for SBO prevention. Some high‐volume institutions (> 300 cases) exhibited SBO rates both above and below the overall mean.

### 3.3 Impact of APM on SBO Incidence

Substantial variation in APM usage was observed across the 28 participating hospitals, with usage rates ranging from 0.0% to 99.2%. Among hospitals with ≥ 20 cases, the median APM usage rate was 67.0% (IQR: 36.4%–91.7%).

In the mixed‐effects logistic regression model, APM use showed no significant association with SBO reduction (Table S1, adjusted OR 1.01, 95% CI 0.78–1.32, *p* = 0.94); accordingly, NNT and ARR were not calculated, as prespecified in the statistical methods. This lack of protective effect remained consistent after adjusting for all covariates including surgical approach, patient demographics, tumor characteristics, and hospital‐level clustering. When stratifying patients by surgical approach, SBO incidence was 4.0% in the laparoscopic APM (+) group and 4.5% in the APM (−) group (*p* = 0.46), and 6.6% versus 7.4% in the corresponding open surgery groups (*p* = 0.48). Thus, APM use showed no significant impact on SBO in either surgical approach. In the cecum, SBO incidence was 2.2% in the APM (+) group and 6.1% in the APM (−) group (*p* = 0.12), whereas no clear differences were observed in other segments. As no site‐specific comparison reached statistical significance, NNT and ARR were not calculated for any tumor location.

### 3.4 The Impact of APM on SBO After Rectal Surgery

A detailed analysis was conducted on the rectum, which had the highest incidence of SBO and the largest number of cases (Table 3). The cases were classified into three groups: no stoma, ileostomy, and colostomy. Further analysis was conducted by dividing them into laparoscopic and open surgery groups; however, no significant differences were observed in any group.

**TABLE 3:** The impact of APM on SBO after rectal surgery.

	APM (+)	APM (−)	*p*
Rectum without stoma	4.9	5.5	0.62
Laparoscopic	4.7	4.3	0.77
Open	5.2	8.1	0.17
Rectum with ileostomy	15.3	8.3	0.35
Laparoscopic	12.0	5.1	0.45
Open	18.0	22.2	0.87
Rectum with colostomy	10.3	10.9	0.85
Laparoscopic	15.0	12.8	0.87
Open	8.4	9.0	0.93

Abbreviation: APM, adhesion prevention material.

### 3.5 The Impact of a Stoma on SBO After Rectal Surgery

Among the rectum group, cases without stoma had a higher laparoscopic surgery rate, less blood loss, shorter surgical time, and a lower incidence of abscess formation compared to cases with stoma. Cases with stoma had a higher frequency of SBO and ileus compared to cases without stoma (Table 4). Cases with ileostomy had a higher laparoscopic surgery rate, a higher use of APM, and less blood loss compared to cases with colostomy. However, there was no difference in the frequency of SBO between cases with colostomy and that with ileostomy (Table 5). Figure 1C presents the overall effect of stoma creation across all colorectal sites, whereas Table 4 provides a focused analysis limited to rectal cancer.

**TABLE 4:** The impact of a stoma on SBO after rectal surgery (with stoma vs without stoma).

	With stoma (*N* = 546)	Without stoma (*N* = 1469)	*p*
Laparoscopic surgery (%)	44.6	61.9	< 0.001
APM (%)	57.6	61.3	0.12
Bleeding (mL)	285	60	< 0.001
Surgical time (min)	335	251	< 0.001
Prophylactic drain (%)	94.1	95.3	0.28
Re‐operation (%)	8.1	6.6	0.25
Abscess formation (%)	9.2	4.6	< 0.001
Ileus (%)	9.7	4.8	< 0.001
SBO (%)	11.4	5.1	< 0.001

Abbreviation: APM, adhesion prevention material.

**TABLE 5:** The impact of a stoma on SBO after rectal surgery (Colostomy VS Ileostomy).

	Colostomy (*N* = 387)	Ileostomy (*N* = 159)	*p*
Laparoscopic surgery (%)	39.9	56.0	< 0.001
APM (%)	73.3	89.9	< 0.001
Bleeding (mL)	350	250	0.004
Surgical time (min)	322	360	0.29
Prophylactic drain (%)	92.0	99.4	0.002
Re‐operation (%)	7.3	10.1	0.27
Abscess formation (%)	10.4	6.3	0.13
Ileus (%)	8.5	12.6	0.15
SBO (%)	10.6	13.2	0.39

Abbreviation: APM, adhesion prevention material.

### 3.6 Multivariate Analysis of Risk Factors for SBO After Colorectal Cancer Surgery

Multivariate analysis demonstrated that open surgery, stoma, re‐operation, and ileus were independent risk factors for SBO after colorectal cancer surgery (Table 6). The effect estimate for stoma was larger in the standard multivariable model (OR 2.52; 95% CI 1.78–3.55) than in the mixed‐effects model (OR 1.84; 95% CI 1.35–2.51), suggesting attenuation after accounting for institutional clustering.

**TABLE 6:** Multivariate analysis of risk factors for SBO after colorectal cancer surgery.

	Odds ratio	95% CI	*p*
Male	1.21	0.93–1.59	0.16
Laparoscopic surgery	0.64	0.48–0.86	0.004
APM	0.86	0.66–1.12	0.27
Stoma	2.52	1.78–3.55	< 0.001
Blood loss	0.95	0.70–1.19	0.75
Operative time	0.92	0.69–1.22	0.57
Re‐operation	6.79	4.76–9.68	< 0.001
Anastomotic leakage	0.91	0.71–1.17	0.47
Ileus	4.32	3.11–6.01	< 0.001

*Note:* This table presents results from the secondary analysis using standard multivariable logistic regression without random effects, performed to identify independent risk factors and to facilitate comparison with previous literature. Note that the odds ratio for laparoscopic surgery in this secondary analysis (OR 0.64; 95% CI 0.48–0.86) differs from that in the primary analysis, which employed mixed‐effects logistic regression accounting for hospital‐level clustering (Table 2; OR 0.58; 95% CI 0.45–0.74).

Abbreviations: APM, adhesion prevention material; 95% CI, 95% confidence interval.

### 3.7 Sensitivity to Unmeasured Confounding

E‐value analysis demonstrated robustness of our primary findings to unmeasured confounding. Laparoscopic surgery had an E‐value of 2.84 (CI limit: 2.04), and stoma creation had an E‐value of 3.08 (CI limit: 2.04), indicating that a confounder would need to be associated with both the exposure and SBO by a risk ratio of at least these magnitudes to fully explain away the observed associations. The *E*‐value for APM was 1.11, consistent with the null finding.

## 4 Discussion

This nationwide survey of 5458 colorectal cancer surgeries provides novel insights into site‐specific risk factors for postoperative SBO. Three key findings were identified. First, laparoscopic surgery was independently associated with a reduced risk of SBO compared with open surgery, with statistically significant reductions observed in ascending colon and sigmoid colon surgery. Second, APMs showed no significant protective effect against SBO in any anatomical subgroup. Third, stoma creation was identified as a major independent risk factor for SBO.

Laparoscopic surgery was associated with a clinically meaningful reduction in SBO risk. While U.S. Clinical Practice Guidelines describe equivalent long‐term oncological outcomes with superior short‐term outcomes [20], our study suggests an important additional long‐term benefit: a potential reduction in long‐term SBO risk. This extends prior observational studies to a larger multicenter cohort with longer follow‐up [3, 9]. The observed variation in SBO incidence across anatomical sites—relatively higher rates in rectal and descending colon surgeries requiring greater retroperitoneal dissection—supports the notion that adhesions arise primarily from visceral rather than parietal peritoneum [21]. Mechanistically, laparoscopy may reduce adhesion formation through less peritoneal trauma and a more favorable intraoperative environment [22], although the specific mechanisms were not evaluated in this study.

The effect of laparoscopy varied according to tumor location. While the overall NNT of 37 represents a clinically meaningful effect, site‐specific analyses revealed important differences across anatomical sites. Statistically significant reductions in SBO risk were observed in ascending colon surgery (NNT = 22.2) and sigmoid colon surgery (NNT = 30.2), whereas no significant reduction was observed in descending colon, transverse colon, cecum, or rectal surgery. In practical terms, performing 22 ascending colon resections laparoscopically instead of open would prevent one case of SBO. These findings suggest that the benefit of minimally invasive surgery is context‐dependent. The absence of effect in transverse colon may reflect its high mobility, complex mesenteric attachments, and frequent need for omental dissection, which diminish the relative advantage of laparoscopy. In rectal surgery, the deep pelvic field, frequent stoma creation, and possible effects of unmeasured factors such as neoadjuvant radiotherapy may also contribute.

APMs had no significant impact on SBO. No statistically significant protective effect was observed in any anatomical subgroup, and this finding remained robust after adjustment for confounders, suggesting limited measurable clinical benefit of APMs in colorectal cancer surgery under current practice patterns. A possible explanation for these findings is that indication bias may have obscured a potential benefit, as surgeons preferentially applied APMs in technically complex or high‐risk operations during the study period. Additionally, Seprafilm application was not standardized across institutions, with usage rates varying dramatically from 0% to 99%. This variability in placement technique, timing, and anatomical coverage may have further attenuated any potential benefit. Our findings align with Saito et al.'s randomized trial, which similarly showed no significant SBO reduction despite APM use in colorectal cancer patients [23]. The discordance with positive results in gynecologic or animal models [24] suggests that prevention of macroscopic adhesions may not translate into prevention of clinically significant SBO, although this hypothesis was not directly tested.

Stoma creation was associated with an approximately 84% increase in SBO risk. This risk was consistent between ileostomy (13.2%) and colostomy (10.6%), indicating that stoma presence itself, rather than type, is the primary determinant. This increased risk may be attributable not only to adhesion formation from the additional peritoneal trauma of stoma creation and subsequent closure [25], but also to non‐adhesive mechanisms such as kinking at the abdominal wall passage or parastomal hernia. The present database did not record the timing of SBO relative to stoma closure; therefore, whether SBO occurred before or after closure could not be determined. The observed increase in SBO risk in stoma patients may thus reflect the cumulative effect of both the primary surgery and the closure procedure, rather than the primary surgery alone. The similarity between ileostomy and colostomy contradicts the assumption that ileostomy carries greater risk due to fluid effluent, suggesting that surgical trauma may be a more important factor than stoma type. These findings underscore the need for judicious stoma creation, reserving it for cases where the risk of anastomotic leakage clearly outweighs the risk of SBO.

Tumor location was an independent determinant of SBO risk. Rectal cancer carried the highest incidence (6.8%), followed by descending colon, while all other colonic sites showed significantly lower odds. This likely reflects both surgical complexity and anatomical constraints. Pelvic surgery involves extensive mesorectal excision in a confined space, often with radiotherapy, stoma creation, and higher inflammatory response, all of which may promote adhesions. Descending colon resections require splenic flexure mobilization and sometimes extension into upper rectum or transverse colon, potentially increasing dissection complexity and small bowel exposure. By contrast, sigmoid colon surgery, though anatomically adjacent, had lower SBO risk, possibly due to more straightforward operative planes and lower stoma rates. These observations highlight the need for site‐specific preventive strategies, especially for rectal cancer surgery.

Several limitations warrant consideration. The retrospective design precludes causal inference, and treatment allocation (laparoscopy vs. open, APM use, stoma creation) was not randomized. Confounding by indication is especially relevant for APM use, as APM may have been preferentially applied in cases with greater surgical complexity, potentially masking a protective effect. Residual confounding from unavailable variables—including tumor stage, body mass index, comorbidities, neoadjuvant therapy, multivisceral resection, peritoneal dissemination, and emergency surgery—cannot be excluded. Notably, these factors share a common directionality: each is associated with a higher likelihood of open surgery and, independently, with an increased risk of postoperative adhesion formation. Consequently, if confounding by indication is present, it would be expected to systematically overestimate the protective effect of laparoscopic surgery on SBO risk. In addition, previous abdominal surgical history and any abdominal surgery performed for other conditions during the follow‐up period were not captured in the dataset. Prior surgery increases the baseline adhesion burden and SBO susceptibility, and may have influenced the choice of surgical approach, representing a potential confounder. Furthermore, SBO events occurring after an intervening abdominal surgery during follow‐up cannot be attributed solely to the index colorectal operation, which may have led to outcome misattribution. Propensity score‐based methods would not resolve this limitation, as adequate covariate data for score construction were unavailable in the present dataset; such analyses might instead provide a false sense of balance. *E*‐value analysis was therefore employed as the prespecified sensitivity analysis to quantify the robustness of our findings to potential unmeasured confounding. However, *E*‐value analysis cannot directly account for or eliminate bias arising from specific unmeasured confounders. The present study quantified the 5‐year risk of postoperative SBO using a binary endpoint (occurrence within five years), as time‐to‐event data were not available in the dataset. This binary approach does not distinguish between early postoperative SBO (which may reflect surgical technique) and late‐onset SBO (which may reflect chronic adhesion maturation), nor does it allow evaluation of the temporal pattern of SBO risk over the follow‐up period. Future studies with time‐to‐event data would enable Kaplan–Meier and Cox regression analyses to better characterize the timing and hazard trajectory of SBO after colorectal cancer surgery. The present database did not allow differentiation between conservatively and surgically managed SBO episodes; therefore, whether the severity of SBO differed between groups could not be assessed. This is an important area for future investigation. Our definition of SBO, although clinically pragmatic, did not require standardized imaging confirmation, and diagnostic criteria may have varied across institutions. This may have led to outcome misclassification, particularly between mechanical SBO and prolonged postoperative ileus [26], as well as inter‐institutional variation in outcome ascertainment. The 5‐year follow‐up, while substantial, may not capture late SBO events that can occur decades after surgery. Moreover, only Seprafilm was available during the study period, limiting generalizability to newer adhesion barriers. Hospital practices varied widely, as evidenced by the funnel plot, and this heterogeneity may affect external validity. Finally, our study period (2012–2014) may not fully reflect current surgical practice. The subsequent adoption of robotic surgery, refined laparoscopic techniques, and ERAS protocols may further reduce peritoneal trauma and could potentially modify the association between minimally invasive surgery and SBO risk. Conversely, the increasing use of diverting ileostomy in sphincter‐preserving rectal surgery may independently influence SBO risk. Accordingly, the magnitude of the association observed in our 2012–2014 cohort may not directly reflect that in contemporary clinical practice. Prospective studies reflecting contemporary surgical practice are needed to confirm our findings.

## 5 Conclusion

Despite these limitations, this study provides important clinical implications for SBO prevention after colorectal cancer surgery. Laparoscopic surgery was associated with a reduced long‐term risk of SBO, with statistically significant benefits observed in ascending and sigmoid colon surgery; however, this association should be interpreted with caution, as residual confounding by unmeasured factors such as BMI, tumor stage, and operative urgency cannot be excluded. Current APM‐based strategies were not significantly associated with a reduction in SBO risk in this study; however, as APM use was at the discretion of individual surgeons without standardized indications, confounding by indication may have obscured a potential protective effect. These findings suggest that minimally invasive approaches may contribute to long‐term SBO prevention in colorectal cancer surgery, although confirmatory studies adjusting for potential confounders are needed. Stoma creation warrants careful consideration because of its associated increase in SBO risk.

## Author Contributions

**Kozo Kataoka:** data curation. **Takeshi Yamada:** conceptualization, methodology, formal analysis, visualization, project administration, writing – original draft, writing – review and editing. **Rai Shimoyama:** data curation. **Yuichi Takayama:** data curation. **Kensuke Kumamoto:** data curation. **Keisuke Ihara:** data curation. **Kiichi Sugimoto:** data curation. **Akihisa Matsuda:** data curation. **Hiromichi Sonoda:** data curation. **Atsuko Fukazawa:** data curation. **Fumihiko Fujita:** data curation. **Akira Komono:** data curation. **Ken Eto:** data curation. **Norio Yukawa:** data curation. **Takuya Nishino:** formal analysis. **Ryo Ohta:** data curation. **Koichi Okuya:** data curation. **Daisuke Ichikawa:** conceptualization. **Yasuyuki Yokoyama:** data curation, formal analysis. **Yasuki Akiyama:** data curation.

## Funding

The authors have nothing to report.

## Ethics Statement

Nippon Medical School Institutional Review Board (Approval No. 29–01‐887).

## Conflicts of Interest

Daisuke Ichikawa is an Editor of Annals of Gastroenterological Surgery. He was not involved in the peer‐review or decision‐making process for this manuscript. The other authors declare no conflicts of interest.

## Supporting information


**Table S1:** Impact of APM on SBO incidence.

## Data Availability

The data that support the findings of this study are available from the corresponding author upon reasonable request.
