# Supplementary material for: Laparoscopic Surgery Is Associated With Reduced Small Bowel Obstruction Risk After Colorectal Cancer Surgery: A Nationwide Cohort Study of 5458 Patients
Source: Ann Gastroenterol Surg. 2026 Sep 10:10.1002/ags3.70280. Online ahead of print. doi: 10.1002/ags3.70280 (PMC13561679; doi:10.1002/ags3.70280)
Supplement: Supplementary file 1 [file AGS3-9999-0-s001.docx]

**Supplement Table S1 Impact of APM on SBO incidence**

|  | APM (+) | APM (-) | OR | 95% C.I. | P |
| --- | --- | --- | --- | --- | --- |
| Total  (N=5458) | 5.2% | 5.2% | 1.01 | 0.78-1.32 | 0.94 |
| Cecum  (N=448) | 2.2% | 6.1% | 0.35 | 0.11-1.13 | 0.12 |
| Ascending colon  (N=953) | 5.5% | 3.4% | 1.64 | 0.85-3.14 | 0.12 |
| Transverse colon  (N=489) | 4.2% | 2.8% | 1.45 | 0.53-3.98 | 0.58 |
| Descending colon  (N=263) | 6.3% | 6.8% | 0.90 | 0.34-2.37 | 0.94 |
| Sigmoid colon  (N=1290) | 3.6% | 4.2% | 0.85 | 0.48-1.48 | 0.57 |
| Rectum  (N=2015) | 6.7% | 6.9% | 0.96 | 0.68-1.37 | 0.56 |

SBO: small bowel obstruction, OR: Odds ratio, 95% C.I.: 95% Confidence Interval, APM: adhesion prevention material
